# *Saccharomyces boulardii* Allows Partial Patients to Avoid Reusing Bismuth Quadruple for *Helicobacter pylori* Rescue Therapy: A Single-Center Randomized Controlled Study

**DOI:** 10.3389/fcimb.2022.903002

**Published:** 2022-07-08

**Authors:** Peng Qu, Xiaoming Liu, Xiujuan Xia, Xiaoran Xie, Ju Luo, Sha Cheng, Jingshu Chi, Peng Liu, Huan Li, Wenfang Zhao, Huihao Yang, Canxia Xu

**Affiliations:** ^1^ Department of Gastroenterology, The Third Xiangya Hospital of Central South University, Changsha, China; ^2^ Hunan Provincial Key Laboratory of Uncontrollable Inflammation and Tumour, The Third Xiangya Hospital of Central South University, Changsha, China

**Keywords:** *Saccharomyces boulardii*, *Helicobacter pylori*, rescue therapy, probiotic, bismuth-based quadruple therapy, eradication

## Abstract

**Background:**

The increasing rate of drug resistance often leads to *Helicobacter pylori* (*H. pylori*) eradication failure and needs the rescue therapy. Thus, the exploration of new rescue therapeutic regimens is important. The present study was designed to test the beneficial effects of *Saccharomyces boulardii* (S.boulardii) prior to *H. pylori* rescue therapy basing on bismuth quadruple.

**Methods:**

One hundred *H. pylori*-infected patients were randomly divided into two groups: study group and control group. Patients in the study group (n=50) underwent two-stages therapy: patients started with S.boulardii monotherapy for 2 weeks, and then tested for *H. pylori* infection after resting for 4 weeks without any therapy, patients who were still positive for *H. pylori* continued with bismuth quadruple eradication therapy. For the control group (n=50), all patients were observed and were not treated with any gastric drugs or antibiotics for 6 weeks, then those who were still positive for *H. pylori* received the same eradication therapy as the study group. Eradication rate, adverse events and the cost-effectiveness of two regimens were analyzed in this study.

**Results:**

The *H.pylori* eradication rate of ITT (intent-to-treat) analysis and PP (per-protocol) analysis in the first phase of treatment were significantly higher in the study group than the control groups respectively (28.0% *vs* 2.0%, *p*<0.001 and 30.4% *vs* 2.1% *p*<0.001). For the total treatment effect, there were no significant differences in the eradication rate of ITT analysis (78.0% *vs* 80.0%) or PP analysis (90.7% *vs* 88.9%) between the study group and the control group. The cost‐effectiveness ratio of the study group was slightly higher than that of the control group (8.95 *vs* 8.55). There were two patients in the study group and four patients in the control group with the adverse events, respectively. There was no significant difference on the incidence of adverse events between the two groups (*p*=0.68).

**Conclusion:**

*S.boulardii* may serve as a beneficial treatment option before *H. pylori* rescue therapy since it callowed partial patients to avoid reusing bismuth quadruple.

## Introduction

*Helicobacter pylori* (*H. pylori*) infection is a common bacterial infection that causes chronic gastritis, peptic ulcers, gastric cancer, MALT lymphoma, and other gastrointestinal diseases (Warren, 2000; Moss and Malfertheiner, 2007; Wang et al., 2014). The prevalence of *H. pylori* is about 50% of the population in China and it has been classified as a class I biologic carcinogen (Ahn and Lee, 2015; Hooi et al., 2017). The bismuth quadruple therapy (proton pump inhibitor plus bismuth and two antibiotics) for two weeks is currently recommended as the main empirical treatment for *H.pylori* eradication (Fallone et al., 2016). However, the development of antibiotic-resistant bacteria and a gradual increase in refractory *H. pylori* infections have been more recently associated with the massive increase in the use of antibiotics, poor patient compliance, and irregular drug use, and the side effects associated with drugs have increased, leading to the development of antibiotic-resistant bacteria and a gradual increase in refractory *H.pylori* infections (Thung et al., 2016; Czerucka and Rampal, 2019). Thus, the exploration of new salvage therapy regimens is important.

To improve the eradication rate and reduce the side effects of drugs, many therapeutic strategies and new drugs have been investigated. High-dose dual therapy and novel acid suppressants blockers such as vonoprazan may contribute to the successful eradication of *H. pylori* through rescue therapy (Shirai et al., 2007; Yang et al., 2015; Hirata et al., 2020; Sue et al., 2021). In addition, several studies have demonstrated that therapeutic regimens are based on antibiotic sensitivity testing (Yahav et al., 2006) as well as drug resistance gene testing (Liou et al., 2013) can improve the effectiveness of salvage therapy, but they are limited by technical issues and the price. Furthermore, Supplementation with polaprezinc (Kashimura et al., 1999), the traditional Chinese medicine (Li et al., 2021), probiotics (Dang et al., 2014), and other drugs are also considered as optional options for the treatment of refractory *H. pylori* infection.

*Saccharomyces boulardii* (S. boulardii),as a probiotics, was first isolated from litchi fruit (Edwards-Ingram et al., 2007). S. boulardii is a non-bacterial microorganism that is resistant to antibiotics, and could partially viable at low pH values. Currently, S. boulardii is mainly used to treat antibiotic-associated diarrhea, Clostridium difficile infections, travelers’ diarrhea, inflammatory bowel diseases, and irritable bowel syndrome, etc (Edwards-Ingram et al., 2007; Szajewska and Kołodziej, 2015). Two meta-analyses have shown that the supplementation with S. boulardii in the standard triple therapy can increased the eradication rate of *H. pylori* and reduced the incidence of overall side effects (Szajewska et al., 2010; Szajewska et al., 2015). However, several studies have reported that treatment with S. boulardii combined with the bismuth quadruple can reduce side effects in patients, but fails to further improve eradication rates (Chen et al., 2018; Zhao et al., 2021; Naghibzadeh et al., 2022). In addition, treatment of *H. pylori* infection with probiotic monotherapy in children has an eradicate 29.3% of cases (Boonyaritichaikij et al., 2009), While treatment with S.boulardii alone has an eradication rate of 11.8% (Gotteland et al., 2005). However, in the previous studies, S.boulardii was limited to the first treatment of *H. pylori* infection or combined with triple therapy or quadruple therapy. In clinical practice, we found that S.boulardii monotherapy eliminated *H. pylori* infection in some patients after multiple treatment failures. Therefore, we designed a randomized controlled clinical trial with an expanded sample size to further evaluate the efficacy and safety of S.boulardii as a pre-treatment for *H. pylori* infection rescue therapy.

## Materials and Methods

### Patients

Patients were enrolled from the department of Gastroenterology at the Third Xiangya Hospital of Central South University. This study had already been approved by The Ethics committee of the Third Xiangya Hospital of Central South University(approval number 21151)and was registered in the national clinical registry NCT05191875. Written informed consent was obtained from all subjects prior to participation.

### Inclusion and Exclusion Criteria

The inclusion criteria were as follows: Participants were aged 18 to 65 years, and both females and males were able to participate; Patients had to have a diagnosis of *H. pylori* infection and have failed treatment for *H. pylori* infection based on the results of the ^13^C or ^14^C-UBT; Patients could not have taken acid-suppressing medications (PPI or H2 blockers) within the last 2 weeks or used antibiotics and/or bismuth within the last 4 weeks; Participants had to understand and be willing to participate in this clinical trial and provide a signed informed consent form.

The Exclusion Criteria were as follows: Participants have a history of drug allergy; severe cardiac, hepatic, pulmonary,or renal insufficiency;a recent history of gastrointestinal hemorrhage, obstruction, perforation, tumors, or other serious organic diseases of the gastrointestinal tract, patients with mental illness, psychological disorders or inability to cooperate with the researchers; or be pregnant, lactating, or planning to have children during the study period.

### Diagnosis for *H. pylori* Infection

A positive *H. pylori* infection was defined as: a positive ^13^C/^14^C urea breath test(^13^C/^14^C-UBT)

^13^C-UBT: The participants fasted for at least 4h, and a breath sample before taking the medicine was taken(pre-dose). The participants took one urea capsule (Beijing Haiderun Pharmaceutical Group Co., Ltd.). After 30 min, a breath sample was taken(post-dose), and then pre-dose and post-dose breath samples were measured using a ^13^C infrared spectrometer. The ^13^C-UBT is usually expressed as a DOB value,where values≥4.0 are taken as positive, and those <4.0 are negative.

^14^C-UBT: Participants were given one capsule of ^14^C-urea breath capsule (Shanghai Xinke Pharmaceutical Co., Ltd.) after fasting from food for at least 3h. The patient blew into the *H. pylori* test breath card (Anhui Yanghe Medical Equipment Co., Ltd.) for about 3~5 min, The cut off value for ^14^C-UBT was taken as 99, >99 dpm/mmol were determined to be positive, while those ≤99 dpm/mmol were determined to be negative.

### Therapeutic Regimens

**Figure 1:**
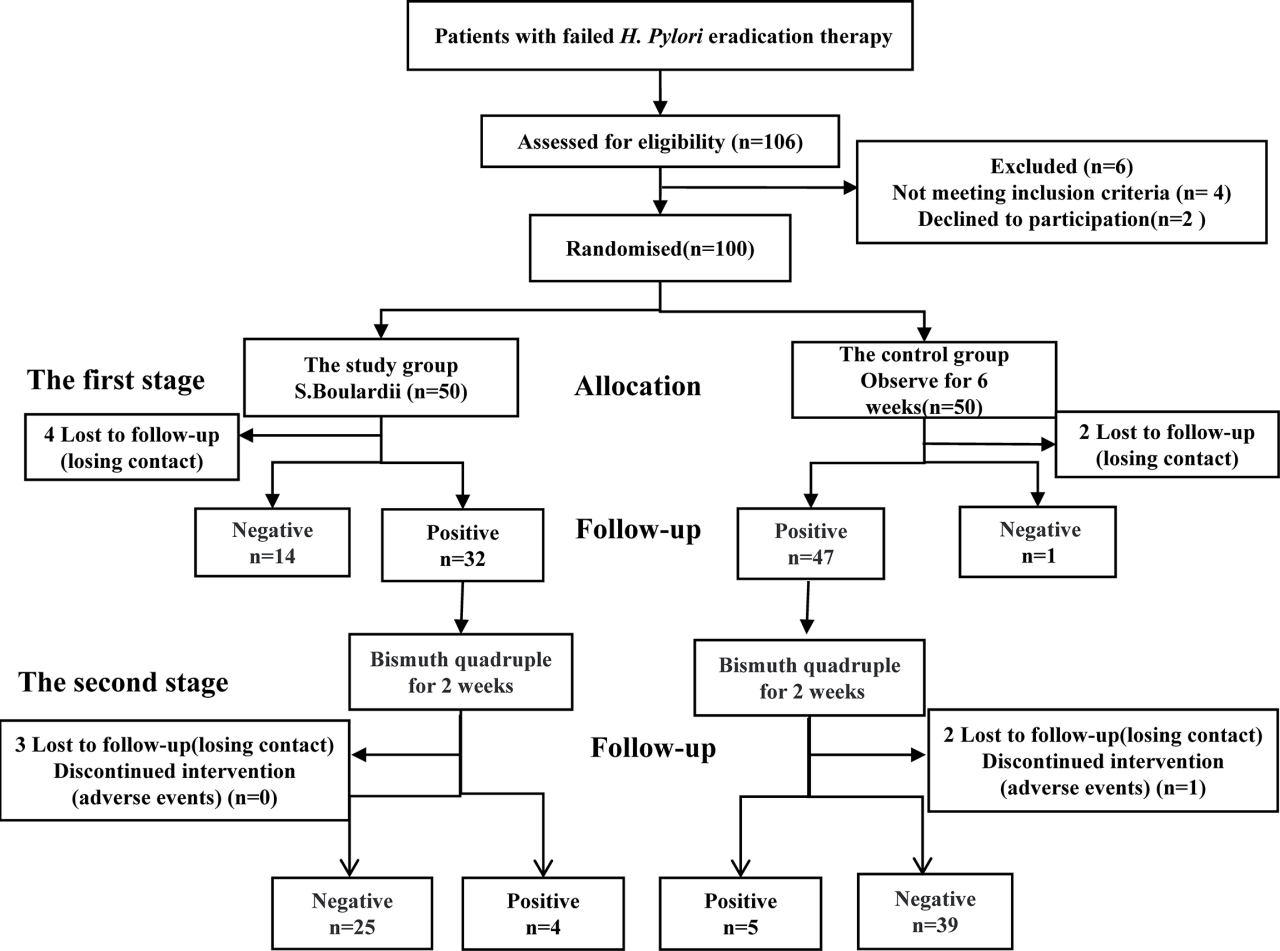
Study design of two therapeutic regimens and flow diagram of the study.

Therapeutic Regimens and study design are illustrated in **(Figure 1)**. This was a single-center, randomized controlled study. Participants were allocated to either the study group or the control group according to a randomization table that was generated by a computerized online random number generator.

The study group (n=50) underwent two stages of treatment. In the first stage, they were treated with S. boulardii (500mg b.i.d) for 14 days. After discontinuation of the drug for at least 4 weeks, *H. pylori* eradication status was confirmed by a positive 13C or 14C-UBT. patients who were still positive for *H.pylori* continued with the second stage of treatment with Ilaprazole(5mg b.i.d), Doxycycline(0.1g b.i.d). Furazolidone(0.1g b.i.d), and Colloidal Bismuth Tartrate **(Figure 1)** (220mg b.i.d) for 14-day therapy as the second stage of treatment.

As for the control study(n=50), patients were observed for 6 weeks without gastric drugs or antibiotics, and then those who were still positive for *H. pylori* received the same eradication therapy as the study group.13C or 14C-UBT was used to evaluate the *H. pylori* eradication rate after at least 4 weeks off drugs **(Figure 1)**.

We analyzed the patients’ clinical data, including the characteristics of the patient, eradication rate, cost-effectiveness, and adverse effects to evaluate the efficacy of S. boulardii as a rescue treatment for *H. pylori* infection. Smoking was defined as at least 1 cigarette/d, continuous or cumulative for ≥6 months, Alcohol is consumed when ≥ 20 g pure alcohol per day. Medication compliance was assessed by the participants’ medication possession ratio (MPR). MPR is the ratio of the actual amount of medication taken by the patient to the amount of medication that should have been taken. MPR>80% was considered as good compliance, and MPR<80% was considered as poor compliance. Patients who did not test ^13^C/^14^C-UBT after treatment were considered to be poor compliance.

### Sample Size Estimation

Pass.15.0 was used to perform the sample size estimation for our study. We used a sample size of 45 for the study group and 45 for the control group, achieving a power level of 90.473% to detect a difference in proportions between groups of 0.23. In our previous clinical observations, we found that S. boulardii alone could achieve an eradication rate of 20-30% during the resuce therapy for *H. pylori*. The proportion in the study group was assumed to be 0.020 under the null hypothesis and 0.25 under the alternative hypothesis. The proportion in the control group was 0.020. The test statistic used was the two-sided Z-Test with pooled variance. The significance level of the test was 0.05. Based on the assumed dropout rate of 10%, we decided to collect 50 patients in each group to verify the effect of the drug treatment.

### Outcomes

The primary outcome of the trial was the eradication rate. All patients were included in the ITT (intent-to-treat) analysis regardless of whether they completed the trial or not. Patients not lost to follow-up and with good medication compliance (MPR>80%) were included in the PP (per-protocol) analysis. The cost of medication per patient only included direct drug costs and breath test cost, effectiveness is the eradication rate in the PP analysis. Patients were followed up by telephone or in the clinic to identify side effects and confirm medication compliance at two and four weeks after taking the medication.

### Statistical Analysis

SPSS.26.0 software was used to analyze the collected data. The radical rate of *H. pylori* was measured by ITT and PP analysis. The measurement data are expressed as the mean ± standard deviation, and the enumeration data are displayed with as percentages. The Student’s t-test was used to compare measurement data between two groups. The Chi-squared or Fisher’s exact test was used for the enumeration data, as appropriate; *p*< 0.05 was considered statistically significant.

## Results

### Characteristics of the Patients

A total of 100 patients who met the inclusion criteria were recruited and were randomly allocated to the study group(n=50) and control groups(n=50). 43 and 45 patients in the two groups completed regimens respectively and attended the follow-up of ^13^C or ^14^C-UBT, respectively. There were no statistical differences in the demographic and clinical characteristics of patients between the two groups(*p*>0.05)(**Table 1**). For the patients in study group, there were no statistical differences in the demographic characteristics of patients between patients who succeeded and failed to eradicate *H. pylori* infection(*p*>0.05)(**Table 2**).

**Table 1:** Demographic characteristics of patients in the first stage of rescue therapy.

Characteristics	The study group	The control group	*p-*value
Patients (n)	50	50	
Age(years,mean ± SD)	44.8± 12.6	47.1± 11.8	0.35
Sex (male/female)	24/26	21/29	0.55
BMI (kg/m2, mean ± SD)	22.67 ± 4.28	22.22 ± 2.85	0.45
Married, n(%)	47 [94.0%]	45 [90.0%]	0.72
Education background (university or above), n (%)	19 [38.0%]	12 [24.0%]	0.13
Disease-associated *H. pylori*, n(%)			
Chronic gastritis, n(%)	33 [55%]	31 [51.7%]	0.68
Gastric polyps, n(%)	6 [12.0%]	3 [6.0%]	0.49
Peptic ulcers, n(%)	6 [12%]	12 [24%]	0.12
Cigarette smoking, n(%)	6 [12.0%]	10 [20.0%]	0.28
Alcohol consumption, n(%)	6 [12.0%]	6 [12.0%]	>0.99
Dietary habit(bland diet),n(%)	29 [58.0%]	30 [60.0%]	0.84
Eating customs (serving chopsticks),n(%)	16 [32.0%]	21 [42.0%]	0.30
Symptom			
Upper abdominal pain,n(%)	16 [32.0%]	23 [46.0%]	0.15
Abdominal distension, n(%)	14 [28.0%]	16 [32.0%]	0.66
Upper abdominal discomfort,n(%)	7 [14.0%]	9 [18.0%]	0.59
Belching,n(%)	10 [20.0%]	12 [24.0%]	0.63
Acid regurgitation,n(%)	5 [10.0%]	9 [18.0%]	0.25
Nausea,n(%)	4 [8.0%]	2 [4.0%]	0.68
No Symptom, n(%)	12 [24.0%]	7 [14.0%]	0.20
Family infection,n(%)	15 [30.0%]	8 [16.0%]	0.10
Times of previous treatment			
1	28 [56.0%]	25 [50.0%]	0.55
≥1	9 [18.0%]	8 [16.0%]	0.79
NA	13	17	
Medication history(regimen)			
Triple therapy	1 [2.0%]	3 [6.0%]	0.62
Quadruple therapy	31 [62.0%]	25 [50.0%]	0.23
Other	0	1 [2.0%]	>0.99
NA	18	21	

**Table 2:** Demographic characteristics of patients who succeeded and failed to achieve eradication in the study group.

Characteristics	The success group	The failure group	*p-*value
Patients (n)	14	32	
Age(years,mean ± SD)	45.2 ± 12.3	44.2 ± 13.1	0.41
Sex (male/female)	6/8	19/13	0.30
BMI (kg/m^2^, mean ± SD)	23.4 ± 3.1	21.9 ± 3.8	0.20
Married, n(%)	13 [92.9%]	30 [93.8%]	1.0
Education background (university or above), n(%)	7 [50.0%]	11 [37.9%]	0.52
Disease-associated *H. pylori*, n(%)			
Chronic gastritis, n(%)	11 [78.6%]	19 [59.4%]	0.32
Gastric polyps, n(%)	0 [0.0%]	1 [3.1%]	1.0
Peptic ulcers, n(%)	2 [14.3%]	4 [12.5%]	1.0
Cigarette smoking, n(%)	1 [7.1%]	4 [12.5%]	1.0
Alcohol consumption, n(%)	2 [14.2%]	3 [9.4%]	1.0
Dietary habit(bland diet), n(%)	10 [83.3%]	16 [50.0%]	0.10
Eating customs (serving chopsticks), n(%)	7 [50.0%]	7 [21.8%]	0.06

### *H. pylori* Eradication Rates

The eradication rate in the first phase of treatment was 28.0% and 2.0% in the ITT analysis and 30.4% and 2.1% in PP analysis for the study group and the control group, respectively(*p*<0.001). For the total treatment effect, the ITT analysis of the eradication rate produced values of 78.0% and 80.0% for the study group and the control group, respectively(*p*=0.81). Values of 90.7% and 88.9% were found for the study and control groups in the PP analysis, respectively (*p*=1.0). There was a significant difference on the eradication rate in the first phase of the two therapeutic regimens, but not for the total and the second phase eradication rates (**Tables 3**–**5**).

**Table 3:** The eradication rate of the first stages of treatment.

Eradication rate	The study group	The control group	*p*-Value
ITT % (n/N)	28.0% (14/50)	2.0% (1/50)	< 0.001
95% CI	15.1-40.9%	-2.0-6.0%	
PP % (n/N)	30.4% (14/46)	2.1% (1/48)	< 0.001
95% confidence interval	16.6-44.2%	-2.1-6.3%	

**Table 4:** The eradication rate of the second stages of treatment.

Eradication rate	The study group	The control group	*p*-Value
ITT % (n/N)	78.1% (25/32)	83.0% (39/47)	0.59
95% CI	63.0-.93.3%	71.8-94.1%	
PP % (n/N)	86.2% (25/29)	88.6% (39/44)	>0.99
95% CI	72.9-99.6%	78.9-98.4%	

**Table 5:** The total eradication rate of the two therapeutic regimens.

Eradication rate	The study group	The control group	*p*-Value
.ITT % (n/N)	78.0% (39/50)	80.0% (40/50)	0.81
95% CI	66.1-89.9%	68.5-91.5%	
PP % (n/N)	90.7% (39/43)	88.9% (40/45)	> 0.99
95% CI	81.7-99.7%	79.3-98.4%	

### The Cost-Effectiveness Analysis

In this study, the average cost per patient is 811.4 yuan for the study group, and 759.9 yuan for the control group(**Table 6**). Cost‐effectiveness analysis also showed that the cost‐effectiveness ratio of the study group (8.95) is slightly higher than the control group (8.55)(**Table 6**).

**Table 6:** The cost-effectiveness analysis of the two therapeutic regimens.

	The study group	The control group
Cost (CNY/patient)	811.4	759.9
Effectiveness (%)	90.7	88.9
Cost-effectiveness ratio	8.95	8.55

### Adverse Events

There were 2 cases of acid reflux or retrosternal discomfort in the study group, while there were 4 cases of nausea, rash, or epigastric discomfort in the control group. There was no statistical difference on the incidence of adverse events between the two groups(*p*=0.68)(**Table 7**). The adverse events were relatively mild and patients recovered on their own after discontinuation of the drug.

**Table 7:** Adverse events of two therapeutic regimens.

Adverse events	The study group (n=50)	The control group (n=50)	*p*-Value
Nausea		2	
Diarrhea			
Constipation			
Acid regurgitation	1		
Allergy			
Rash		1	
Upper abdominal discomfort		1	
Posterior sternal discomfort	1		
Anorexia			
Dizziness			
Overall % (n/N)	4% (2/50)	8% (4/50)	0.68
Poor compliance (MPR>80%) n, (%)	0% (0/50)	2.0% (1/50)	1.0

## Discussion

Our study indicates that the two therapeutic regimens we adopted both achieved satisfactory *H. pylori* eradication rates and that S. boulardii can have an inhibitory effect on *H. pylori* to some extent. S.boulardii may be used as a pre-treatment of rescue therapy for *H. pylori* infection.

According to the AGA(American Gastroenterological Association) Expert Review on Clinical practice for refractory *H. pylori* Infection, one and more failures are enough to define refractory *H. pylori* infection (Shah et al., 2021). Thus, cases with one or more failed *H. pylori* treatments, we should use drugs with caution. The Maastricht V/Florence Consensus states that fluoroquinolone containing triple or quadruple therapy can be recommended for *H. pylori*-infected patients with failure by bismuth quadruple therapy (Malfertheiner et al., 2017). However, some patients still do not achieve successful eradication of their diseases due to antibiotic resistance and the lack of patient compliance.

After treatment failure, *H. pylori* sphericity and biofilm formation are in the dormant stage, and continuous intensive antibiotic treatment is not recommended (Tshibangu-Kabamba and Yamaoka, 2021). Bacteria are more sensitive to antibiotics when they are in a relatively active reproductive state (Camacho Mateu et al., 2021). Several studies have demonstrated that continuous treatment with long courses of antibiotics could not only lead to the selection of drug-resistant bacteria (Jakobsson et al., 2007) but could even lead to serious drug side effects such as severe diarrhea and Clostridium difficile infection (Nei et al., 2020). Recent studies have also shown that *H. pylori* treatment led to dysbiosis of Stomach microbiota, while probiotic supplementation could restore the disturbed stomach microbiota in some patients (Yuan et al., 2021). Therefore,we applied S. boulardii alone before the next treatment. The resistance rates of doxycycline and furazolidone in China are 9.2% and 1.49%, respectively (Shao et al., 2018). For rescue therapeutic drugs, a study shows that bismuth quadruple containing doxycycline and furazolidone had a cure rate of more than 90% (Zhou et al., 2020). Therefore, these two antibiotics were used as rescue treatments for *H. pylori* in our study. The result of our study showed no statistical difference in *H. pylori* eradication rates between the two treatment regimens,which may be explained by the already strong therapeutic effect of the bismuth quadruple containing doxycycline furazolidone, In addtion, small sample and the long intermittent period between first stage and second stage may also affect the anti-H. pylori treatment.

To the best of our knowledge, this is the first study to use S. boulardii alone to treat patients who have failed *H. pylori* treatment. Several studies have proposed mechanisms for the probiotic eradication of *H.pylori* infection including protection of the mucosal barrier, the secretion of antimicrobials, modulation of the immune response, and co-aggregation and aggregation (Qureshi et al., 2019). As for S. boulardii, the current research shows that S. boulardii can eradicate *H. pylori* infection through a variety of mechanisms *in vitro* and *in vivo*. Sakarya S et al. reported that S.boulardii prevented the binding of surface α(2-3)-linked sialic acid to the ligand of sialic acid-binding *H. pylori* adhesin, which in turn inhibited the adhesion of *H. pylori* to duodenal epithelial cells (Sakarya and Gunay, 2014). In addition, S. boulardii can exerted an immunoprotective effect by stimulating sIgA and immunoglobulin secretion in the gastrointestinal tract (Buts et al., 1990).

For clinical research, it has been reported that the eradication rate of *H. pylori* infection in children when S. boulardii alone is used for the first treatment is 11.8% (Gotteland et al., 2005). Our study found that S.boulardii was able to achieve an eradication rate of 28.0% during the resuce therapy. There are some reasons for this, as follows: Firstly, the dose and usage of S. boulardii we applied differed from those used in the previous study; Secondly, the rescue treatment was different from the first treatment, and we speculate that only some drug-resistant bacteria survived after the previous treatment, but S. boulardii has a different mechanism from antibiotics and can remove the antibiotic-resistant *H. pylori*. Thirdly, the sensitivity and specificity of the ^13^C or ^14^C-UBT instrument varies from company to company. Notably, 2% of controls were eradicated without any medication, which may be a false positive or false negative ^13^C/^14^C breath test.

S. boulardii is relatively safe and does not colonize the human gastrointestinal tract. It only results in a transient effect on the intestinal flora when taken and few people had side effects in our study. S.boulardii is a fungus that is partially viable at low pH levels and is highly tolerant to bile acids (Edwards-Ingram et al., 2007). The optimal growth temperature for S. boulardii is approximately 37 °C, which is similar to human body temperature (Graff et al., 2008). S. boulardii changes in the gut microbiota and thus reduces adverse gastrointestinal reactions in patients (Cárdenas et al., 2020). The most severe adverse effect of S. boulardii is fungemia, which has been reported mostly in preterm infants and critically ill patients, and has a low incidence rate (Roy et al., 2017; Rannikko et al., 2021). However, there was no statistical differences in adverse effects between the two therapeutic regimens tested in the present study, which may be related to our relatively small sample size. Salvage therapy options for treating *H. pylori* infection with amoxicillin allergy are relatively rare (Dutta and Phull, 2021). We may be able to improve the eradication rate of *H. pylori* by using S. boulardii as a pretreatment before the next treatment. Based on the safety of S. boulardii, it may also be more suitable for older people as well as children with *H. pylori* infection.

There are some limitations to this study. First, this study was a single-center controlled study that needs to be validated in different countries and regions. In addition, the small sample size and high dropout rate may have had some influence on the results. Furthermore, the use of blinded methods as well as placebo controls would further improve the accuracy of the findings.

## Conclusion

The efficacy of both two regimens was satisfactory during rescue therapy for *H. pylori* eradication. S. boulardii monotherapy affects refractory *H. pylori* infection and partially cures *H. pylori* infection before the next bismuth quadruple therapy.Some patients who are successfully treated with S. boulardii will prevent patients from reusing bismuth quadruple. The precise mechanism of the treatment of *H.pylori* by S. boulardii, dosing schedule, and dosage remain to be further studied, and more studies are needed to explore new rescue therapy regimens.

## Data Availability Statement

The raw data supporting the conclusions of this article will be made available by the authors, without undue reservation.

## Ethics Statement

This study was reviewed and approved by IRB of The Third Xiangya Hospital of Central South University. The patients/participants provided their written informed consent to participate in this study.

## Author Contributions

PQ and CX designed the study, XL, XJXA, and XX, recruited the patients and collected clinical data, PL , JC, and WZ followed up with the patients, HL and HY analyzed the data, JL and SC prepared the ethical materials, PQ wrote the article, and all authors participated in the revision of the manuscript. All authors contributed to the article and approved the submitted version.

## Funding

This work was supported by the following grants and foundations: National Natural Science Foundation of China (No. 81570509) ,Changsha Municipal Natural Science Foundation Project (kq2202118) and Hunan Provincial Natural Science Foundation Project(S2022JJMSXM2919).

## Conflict of Interest

The authors declare that the research was conducted in the absence of any commercial or financial relationships that could be construed as a potential conflict of interest.

## Publisher’s Note

All claims expressed in this article are solely those of the authors and do not necessarily represent those of their affiliated organizations, or those of the publisher, the editors and the reviewers. Any product that may be evaluated in this article, or claim that may be made by its manufacturer, is not guaranteed or endorsed by the publisher.

## Abbreviations

*H. pylori*, Helicobacter pylori; S.boulardii, *Saccharomyces boulardii*; SD, standard deviation; ITT Analysis, Intention-to-treat analysis; PP Analysis, Per-protocol analysis; CI, confidence interval.

## References

[B1] Ahn H. J. Lee D. S. (2015). Helicobacter Pylori in Gastric Carcinogenesis. World J. Gastrointest. Oncol. 7 (12), 455–465. doi: 10.4251/wjgo.v7.i12.455 26690981 PMC4678392

[B2] Boonyaritichaikij S. Kuwabara K. Nagano J. Kobayashi K. Koga Y. (2009). Long-Term Administration of Probiotics to Asymptomatic Pre-School Children for Either the Eradication or the Prevention of Helicobacter Pylori Infection. Helicobacter 14 (3), 202–207. doi: 10.1111/j.1523-5378.2009.00675.x 19702850

[B3] Buts J. P. Bernasconi P. Vaerman J. P. Dive C. (1990). Stimulation of Secretory IgA and Secretory Component of Immunoglobulins in Small Intestine of Rats Treated With Saccharomyces Boulardii. Dig. Dis. Sci. 35 (2), 251–256. doi: 10.1007/bf01536771 2302983

[B4] Camacho Mateu J. Sireci M. Muñoz M. A. (2021). Phenotypic-Dependent Variability and the Emergence of Tolerance in Bacterial Populations. PloS Comput. Biol. 17 (9), e1009417. doi: 10.1371/journal.pcbi.1009417 34555011 PMC8492070

[B5] Cárdenas P. A. Garcés D. Prado-Vivar B. Flores N. Fornasini M. Cohen H.. (2020). Effect of Saccharomyces Boulardii CNCM I-745 as Complementary Treatment of Helicobacter Pylori Infection on Gut Microbiome. Eur. J. Clin. Microbiol. Infect. Dis. 39 (7), 1365–1372. doi: 10.1007/s10096-020-03854-3 32125555

[B6] Chen L. Xu W. Lee A. He J. Huang B. Zheng W.. (2018). The Impact of Helicobacter Pylori Infection, Eradication Therapy and Probiotic Supplementation on Gut Microenvironment Homeostasis: An Open-Label, Randomized Clinical Trial. EBioMedicine 35, 87–96. doi: 10.1016/j.ebiom.2018.08.028 30145102 PMC6161473

[B7] Czerucka D. Rampal P. (2019). Diversity of Saccharomyces Boulardii CNCM I-745 Mechanisms of Action Against Intestinal Infections. World J. Gastroenterol. 25 (18), 2188–2203. doi: 10.3748/wjg.v25.i18.2188 31143070 PMC6526157

[B8] Dang Y. Reinhardt J. D. Zhou X. Zhang G. (2014). The Effect of Probiotics Supplementation on Helicobacter Pylori Eradication Rates and Side Effects During Eradication Therapy: A Meta-Analysis. PloS One 9 (11), e111030. doi: 10.1371/journal.pone.0111030 25365320 PMC4217763

[B9] Dutta A. K. Phull P. S. (2021). Treatment of Helicobacter Pylori Infection in the Presence of Penicillin Allergy. World J. Gastroenterol. 27 (44), 7661–7668. doi: 10.3748/wjg.v27.i44.7661 34908805 PMC8641050

[B10] Edwards-Ingram L. Gitsham P. Burton N. Warhurst G. Clarke I. Hoyle D.. (2007). Genotypic and Physiological Characterization of Saccharomyces Boulardii, the Probiotic Strain of Saccharomyces Cerevisiae. Appl. Environ. Microbiol. 73 (8), 2458–2467. doi: 10.1128/aem.02201-06 17293506 PMC1855594

[B11] Fallone C. A. Chiba N. van Zanten S. V. Fischbach L. Gisbert J. P. Hunt R. H.. (2016). The Toronto Consensus for the Treatment of Helicobacter Pylori Infection in Adults. Gastroenterology 151 (1), 51–69.e14. doi: 10.1053/j.gastro.2016.04.006 27102658

[B12] Gotteland M. Poliak L. Cruchet S. Brunser O. (2005). Effect of Regular Ingestion of Saccharomyces Boulardii Plus Inulin or Lactobacillus Acidophilus LB in Children Colonized by Helicobacter Pylori. Acta Paediatr. 94 (12), 1747–1751. doi: 10.1111/j.1651-2227.2005.tb01848.x 16421034

[B13] Graff S. Chaumeil J. C. Boy P. Lai-Kuen R. Charrueau C. (2008). Influence of pH Conditions on the Viability of Saccharomyces Boulardii Yeast. J. Gen. Appl. Microbiol. 54 (4), 221–227. doi: 10.2323/jgam.54.221 18802321

[B14] Hirata Y. Yamada A. Niikura R. Shichijo S. Hayakawa Y. Koike K. (2020). Efficacy and Safety of a New Rifabutin-Based Triple Therapy With Vonoprazan for Refractory Helicobacter Pylori Infection: A Prospective Single-Arm Study. Helicobacter 25 (5), e12719. doi: 10.1111/hel.12719 32602161

[B15] Hooi J. K. Y. Lai W. Y. Ng W. K. Suen M. M. Y. Underwood F. E. Tanyingoh D.. (2017). Global Prevalence of Helicobacter Pylori Infection: Systematic Review and Meta-Analysis. Gastroenterology 153 (2), 420–429. doi: 10.1053/j.gastro.2017.04.022 28456631

[B16] Jakobsson H. Wreiber K. Fall K. Fjelstad B. Nyrén O. Engstrand L. (2007). Macrolide Resistance in the Normal Microbiota After Helicobacter Pylori Treatment. Scand. J. Infect. Dis. 39 (9), 757–763. doi: 10.1080/00365540701299608 17701712

[B17] Kashimura H. Suzuki K. Hassan M. Ikezawa K. Sawahata T. Watanabe T.. (1999). Polaprezinc, a Mucosal Protective Agent, in Combination With Lansoprazole, Amoxycillin and Clarithromycin Increases the Cure Rate of Helicobacter Pylori Infection. Aliment. Pharmacol. Ther. 13 (4), 483–487. doi: 10.1046/j.1365-2036.1999.00510.x 10215732

[B18] Li Y. Li X. Tan Z. (2021). An Overview of Traditional Chinese Medicine Therapy for Helicobacter Pylori-Related Gastritis. Helicobacter 26 (3), e12799. doi: 10.1111/hel.12799 33765344

[B19] Liou J. M. Chen C. C. Chang C. Y. Chen M. J. Fang Y. J. Lee J. Y.. (2013). Efficacy of Genotypic Resistance-Guided Sequential Therapy in the Third-Line Treatment of Refractory Helicobacter Pylori Infection: A Multicentre Clinical Trial. J. Antimicrob. Chemother. 68 (2), 450–456. doi: 10.1093/jac/dks407 23099849

[B20] Malfertheiner P. Megraud F. O'Morain C. A. Gisbert J. P. Kuipers E. J. Axon A. T.. (2017). Management of Helicobacter Pylori Infection-the Maastricht V/Florence Consensus Report. Gut 66 (1), 6–30. doi: 10.1136/gutjnl-2016-312288 27707777

[B21] Moss S. F. Malfertheiner P. (2007). Helicobacter and Gastric Malignancies. Helicobacter 12 Suppl 1, 23–30. doi: 10.1111/j.1523-5378.2007.00539.x 17727457

[B22] Naghibzadeh N. Salmani F. Nomiri S. Tavakoli T. (2022). Investigating the Effect of Quadruple Therapy With Saccharomyces Boulardii or Lactobacillus Reuteri Strain (DSMZ 17648) Supplements on Eradication of Helicobacter Pylori and Treatments Adverse Effects: A Double-Blind Placebo-Controlled Randomized Clinical Trial. BMC Gastroenterol. 22 (1), 107. doi: 10.1186/s12876-022-02187-z 35255819 PMC8903632

[B23] Nei T. Hagiwara J. Takiguchi T. Yokobori S. Shiei K. Yokota H.. (2020). Fatal Fulminant Clostridioides Difficile Colitis Caused by Helicobacter Pylori Eradication Therapy; a Case Report. J. Infect. Chemother. 26 (3), 305–308. doi: 10.1016/j.jiac.2019.10.021 31822448

[B24] Qureshi N. Li P. Gu Q. (2019). Probiotic Therapy in Helicobacter Pylori Infection: A Potential Strategy Against a Serious Pathogen? Appl. Microbiol. Biotechnol. 103 (4), 1573–1588. doi: 10.1007/s00253-018-09580-3 30610283

[B25] Rannikko J. Holmberg V. Karppelin M. Arvola P. Huttunen R. Mattila E.. (2021). Fungemia and Other Fungal Infections Associated With Use of Saccharomyces Boulardii Probiotic Supplements. Emerg. Infect. Dis. 27 (8), 2090–2096. doi: 10.3201/eid2708.210018 PMC8314839 34287140

[B26] Roy U. Jessani L. G. Rudramurthy S. M. Gopalakrishnan R. Dutta S. Chakravarty C.. (2017). Seven Cases of Saccharomyces Fungaemia Related to Use of Probiotics. Mycoses 60 (6), 375–380. doi: 10.1111/myc.12604 28133894

[B27] Sakarya S. Gunay N. (2014). Saccharomyces Boulardii Expresses Neuraminidase Activity Selective for α2,3-Linked Sialic Acid That Decreases Helicobacter Pylori Adhesion to Host Cells. Apmis 122 (10), 941–950. doi: 10.1111/apm.12237 24628732

[B28] Shah S. C. Iyer P. G. Moss S. F. (2021). AGA Clinical Practice Update on the Management of Refractory Helicobacter Pylori Infection: Expert Review. Gastroenterology 160 (5), 1831–1841. doi: 10.1053/j.gastro.2020.11.059 33524402 PMC8281326

[B29] Shao Y. Lu R. Yang Y. Xu Q. Wang B. Ye G. (2018). Antibiotic Resistance of Helicobacter Pylori to 16 Antibiotics in Clinical Patients. J. Clin. Lab. Anal. 32 (4), e22339. doi: 10.1002/jcla.22339 28984385 PMC6817019

[B30] Shirai N. Sugimoto M. Kodaira C. Nishino M. Ikuma M. Kajimura M.. (2007). Dual Therapy With High Doses of Rabeprazole and Amoxicillin Versus Triple Therapy With Rabeprazole, Amoxicillin, and Metronidazole as a Rescue Regimen for Helicobacter Pylori Infection After the Standard Triple Therapy. Eur. J. Clin. Pharmacol. 63 (8), 743–749. doi: 10.1007/s00228-007-0302-8 17565490

[B31] Sue S. Sasaki T. Kaneko H. Irie K. Kondo M. Maeda S. (2021). Helicobacter Pylori Rescue Treatment With Vonoprazan, Metronidazole, and Sitafloxacin in the Presence of Penicillin Allergy. JGH Open 5 (2), 307–311. doi: 10.1002/jgh3.12492 33553672 PMC7857288

[B32] Szajewska H. Horvath A. Kołodziej M. (2015). Systematic Review With Meta-Analysis: Saccharomyces Boulardii Supplementation and Eradication of Helicobacter Pylori Infection. Aliment. Pharmacol. Ther. 41 (12), 1237–1245. doi: 10.1111/apt.13214 25898944

[B33] Szajewska H. Horvath A. Piwowarczyk A. (2010). Meta-Analysis: The Effects of Saccharomyces Boulardii Supplementation on Helicobacter Pylori Eradication Rates and Side Effects During Treatment. Aliment. Pharmacol. Ther. 32 (9), 1069–1079. doi: 10.1111/j.1365-2036.2010.04457.x 21039671

[B34] Szajewska H. Kołodziej M. (2015). Systematic Review With Meta-Analysis: Saccharomyces Boulardii in the Prevention of Antibiotic-Associated Diarrhoea. Aliment. Pharmacol. Ther. 42 (7), 793–801. doi: 10.1111/apt.13344 26216624

[B35] Thung I. Aramin H. Vavinskaya V. Gupta S. Park J. Y. Crowe S. E.. (2016). Review Article: The Global Emergence of Helicobacter Pylori Antibiotic Resistance. Aliment. Pharmacol. Ther. 43 (4), 514–533. doi: 10.1111/apt.13497 26694080 PMC5064663

[B36] Tshibangu-Kabamba E. Yamaoka Y. (2021). Helicobacter Pylori Infection and Antibiotic Resistance - From Biology to Clinical Implications. Nat. Rev. Gastroenterol. Hepatol. 18 (9), 613–629. doi: 10.1038/s41575-021-00449-x 34002081

[B37] Wang F. Meng W. Wang B. Qiao L. (2014). Helicobacter Pylori-Induced Gastric Inflammation and Gastric Cancer. Cancer Lett. 345 (2), 196–202. doi: 10.1016/j.canlet.2013.08.016 23981572

[B38] Warren J. R. (2000). Gastric Pathology Associated With Helicobacter Pylori. Gastroenterol. Clin. North Am. 29 (3), 705–751. doi: 10.1016/s0889-8553(05)70139-4 11030082

[B39] Yahav J. Samra Z. Niv Y. Evans C. T. Passaro D. J. Dinari G.. (2006). Susceptibility-Guided vs. Empiric Retreatment of Helicobacter Pylori Infection After Treatment Failure. Dig. Dis. Sci. 51 (12), 2316–2321. doi: 10.1007/s10620-006-9302-2 17078005

[B40] Yang J. C. Lin C. J. Wang H. L. Chen J. D. Kao J. Y. Shun C. T.. (2015). High-Dose Dual Therapy is Superior to Standard First-Line or Rescue Therapy for Helicobacter Pylori Infection. Clin. Gastroenterol. Hepatol. 13 (5), 895–905.e895. doi: 10.1016/j.cgh.2014.10.036 25460556 PMC4404168

[B41] Yuan Z. Xiao S. Li S. Suo B. Wang Y. Meng L.. (2021). The Impact of Helicobacter Pylori Infection, Eradication Therapy, and Probiotics Intervention on Gastric Microbiota in Young Adults. Helicobacter 26 (6), e12848. doi: 10.1111/hel.12848 34448282

[B42] Zhao Y. Yang Y. Aruna Xiao J. Song J. Huang T.. (2021). Saccharomyces Boulardii Combined With Quadruple Therapy for Helicobacter Pylori Eradication Decreased the Duration and Severity of Diarrhea: A Multi-Center Prospective Randomized Controlled Trial. Front. Med. (Lausanne) 8. doi: 10.3389/fmed.2021.776955 PMC8637152 34869495

[B43] Zhou J. J. Shi X. Zheng S. P. Tang D. Cai T. Yao Y.. (2020). Efficacy of Bismuth-Based Quadruple Therapy for Eradication of Helicobacter Pylori Infection Based on Previous Antibiotic Exposure: A Large-Scale Prospective, Single-Center Clinical Trial in China. Helicobacter 25 (6), e12755. doi: 10.1111/hel.12755 32914914

